# Supplementary material for: Effects of Vaccination against Recombinant FSH or LH Receptor Subunits on Gonadal Development and Functioning Male Rats
Source: Vet Sci. 2024 Apr 15;11(4):176. doi: 10.3390/vetsci11040176 (PMC11054695; doi:10.3390/vetsci11040176)
Supplement: Supplementary file 1 [file vetsci-11-00176-s001.zip › vetsci-2880150-supplementary.pdf]

Effects of Vaccination against Recombinant FSH or LH Receptor Subunits on Gonadal Development and Functioning Male Rats

Fuqiang Pan [1], Wanzhen Fu [1], Bochao Zhang [1], Mengdi Han [1], Huihui Xie [1], Qing Yi [1], Wei Qian [1], Jiankun Cui [1], Meng Cao [1], Yanqiuhong Li [1], Yuke Jia [1], Fugui Fang [1,2,3], Yinghui Ling [1,2,3], Yunsheng Li [1,2,3] and Ya Liu [1,2,3,*]

1 Departmet of Veterinary Medicine, College of Animal Science and Technology, Anhui Agricultural University, 130 Changjiang West Road, Hefei 230036, China; 17356582644@163.com (F.P.); fwz@stu.ahau.edu.cn (W.F.); 15155931671@163.com (B.Z.); hanmengdi4017@163.com (M.H.); xhui2013@163.com (H.X.); 18855420135@163.com (Q.Y.); qw2230795053@gmail.com (W.Q.); pecodr1114@163.com (J.C.); 16632238281@163.com (M.C.); 18380512787@163.com (Y.L.); 13389161914@163.com (Y.J.); fgfang@ahau.edu.cn (F.F.); lingyinghui@ahau.edu.cn (Y.L.); lys@ahau.edu.cn (Y.L.)

2 Anhui Provinciale Key Laboratory of Local Livestock and Poultry, Genetical Resource Conservation and Breeding, College of Animal Science and Technology, Anhui Agricultural University, Hefei 230036, China

3 Linquan County Modern Agriculture Technology Cooperation and Extension Service Center, Fuyang 236000, China

* Correspondence: liuya@ahau.edu.cn

Supplementary Materials

Supplementary Table S1

Screening of antigenic epitopes

Group	Selected antigenic epitopes
FSHR	35-45、201-210
LHR	79-89、257-266

Table S1 note: FSHR finally selected sequences 35-45 and 201-210 for subsequent experiments. LHR finally selected sequences 79-89 and 257-266 for subsequent experiments.

Supplementary Figure S1

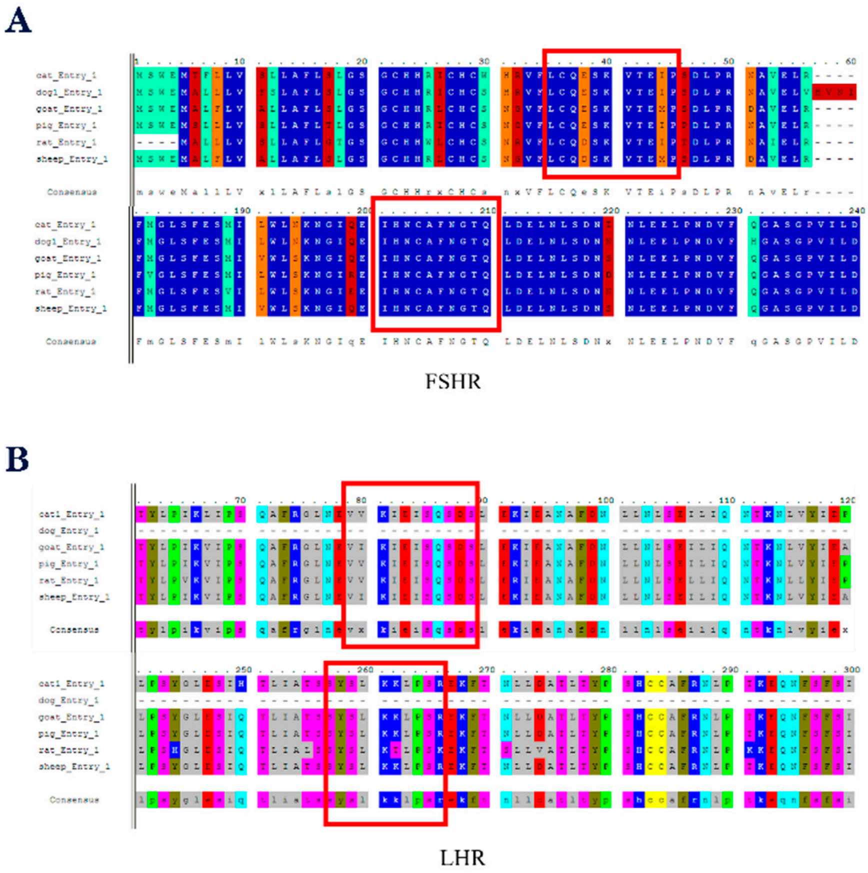

Supplementary Figure S1. Conservative comparison.

Figure S1 note： The conservation of FSHR and LHR protein fragments were compared with sequences from dogs, rats, sheep, goats, and cats. A indicates that the FSHR fragments 35-45 and 201-210 are relatively conserved among multiple species. B indicates that LHR fragments 79-89 and 257-266 are relatively conserved among multiple species. The red box represents the selected sequence.

Supplementary Figure S2

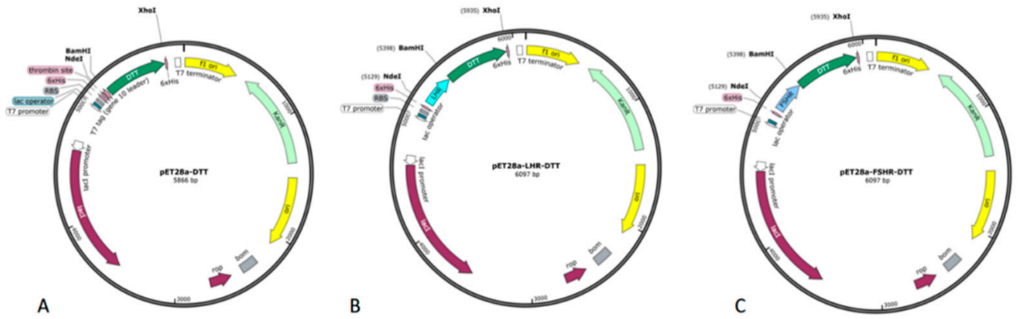

Supplementary Figure S2. pET28a recombinant plasmid map.

Figure S2 note ： The recombinant plasmid map of the pET28a series used in this experiment. A indicates the pET28a-DTT recombinant plasmid, which is used to immunize NC group rats after expressing the protein. B indicates the pET28a-LHR-DTT recombinant plasmid, which is used to immunize LHR group rats after expressing the protein. C indicates the pET28a-FSHR-DTT recombinant plasmid, which expresses the protein and is used to immunize the FSHR group rats.

Supplementary Figure S3

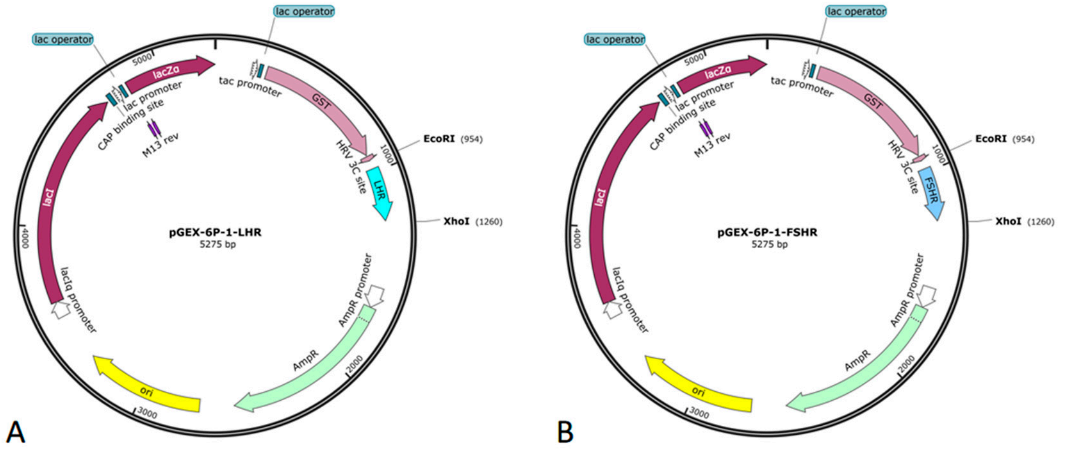

Supplementary Figure S3. pGEX-6P-1 recombinant plasmid map.

Figure S3 note： The recombinant plasmid map. A indicates pGEX-6p-1-LHR, which is used as a coating protein for ELISA detection of LHR antibodies. B indicates pGEX-6p-1-FSHR, which is used as a coating protein for ELISA detection of FSHR antibodies.

Supplementary Figure S4

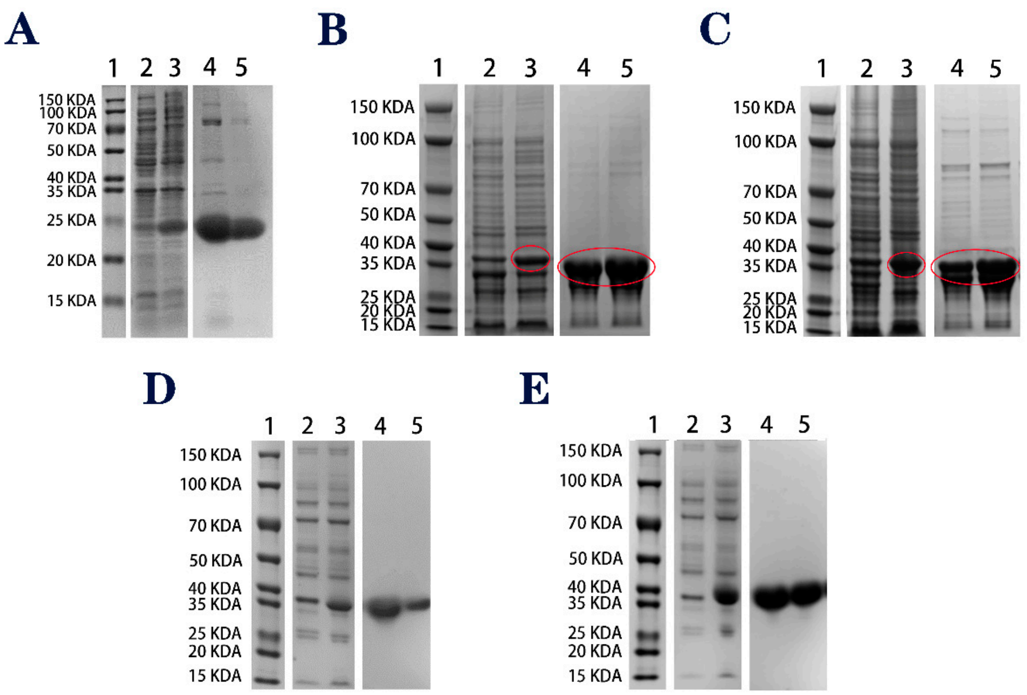

Supplementary Figure S4. Results of purification induced by the coated protein.

Figure S4 note: After induction by IPTG, significant protein bands appeared at the theoretical size, and a large number of single proteins also appeared at the theoretical size in the final elution solution.

As shown in the figure, the expression and purification of the target protein were successful, and subsequent experiments were conducted. A. HIS-DTT（23.5 KDA）, B. HIS-FSHR-DTT（32 KDA）, C. HIS-LHR-DTT（32.5 KDA）, D. GST-FSHR（36.6 KDA）, E.GST-LHR（37.6 KDA）; Lane 1: marker; Lane 2: before induction; Lane 3: induced; Lane 4: elution 1; Lane 5: elution 2. Red circle: target protein.
